# Factors associated with the perception of inadequate sanitary control in 12 Latin American countries during the COVID-19 pandemic

**DOI:** 10.3389/fpubh.2022.934087

**Published:** 2022-07-25

**Authors:** Christian R. Mejia, Daniela Liendo-Venegas, Fernanda García-Gamboa, Miguel A. Mejía-Rodríguez, Mario J. Valladares-Garrido

**Affiliations:** ^1^Secretaría de Doctorado y Posdoctorado de la Facultad de Ciencias Económicas, Universidad de Buenos Aires, Buenos Aires, Argentina; ^2^Facultad de Ciencias de la Empresa, Universidad Continental, Huancayo, Peru; ^3^Facultad de Ciencias de la Salud, Escuela de Medicina Humana de la Universidad Privada de Tacna, Tacna, Peru; ^4^Facultad de Medicina Humana “Manuel Huamán Guerrero” de la Universidad Ricardo Palma, Lima, Peru; ^5^Facultad de Medicina y Cirugía, Universidad Católica de Honduras, Campus San Pedro y San Pablo, San Pedro Sula, Honduras; ^6^Vicerrectorado de Investigación, Universidad Privada Norbert Wiener, Lima, Peru; ^7^Oficina de Epidemiología, Hospital Regional Lambayeque, Lambayeque, Peru

**Keywords:** coronavirus, COVID-19, government, public health, Latin America

## Abstract

**Introduction:**

Sanitary control mechanisms differ greatly from country to country. Therefore, it is important to know citizens' perception of different realities. We aimed to determine the factors associated with the perception of inadequate sanitary control in 12 Latin American countries during the COVID-19 pandemic.

**Methods:**

This is an analytical cross-sectional study. We asked about six perceptions in regard to different situations experienced by inhabitants of 12 Latin American countries during the pandemic. Frequencies according to country were described and associations vs. other important variables were obtained.

**Results:**

Out of 8,489 participants, 68% stated that there were moments of collective hysteria. Honduras was the country that most perceived inadequate control mechanisms established by the government. Multivariate analysis showed that there were statistically significant differences among many of the countries according to the six evaluated items. The higher the level of education, the greater the perception of poor control in five of the aspects. Additionally, men had a lower perception of inadequate control. The older the age, the lower the perception of inadequate control regarding whether there was collective hysteria and shortages of basic essentials. Those with COVID-19 had a lower perception of medicine shortages.

**Conclusion:**

The population of multiple realities in Latin America have perceived a bad management of the pandemic. Citizens' perception is an important indicator of the performance of each government during the COVID-19 pandemic. This study may provide valuable information on the relationship between the effectiveness of government sanitary control and people's mental health, which ultimately helps to create objective prevention programs against post-traumatic stress disorder, depression, fear of contagion, and collective hysteria. In addition, governments could use this information to design effective mitigation plans for future unavoidable pandemic events based on the six criteria discussed here.

## Introduction

The COVID-19 pandemic caused many problems in various aspects of society (1). Something that serious had not been seen since the Spanish Flu (2). Several governments did not have a plan to face a pandemic and very few countries in the world had foreseen that (such as Germany and the USA). However, it generated serious havoc even in those first world countries.

If these developed countries, which had plans to deal with pandemics, were greatly affected, it is not necessary to highlight the great suffering of the developing countries of our continent (3). Moreover, Peru is even recognized as the most affected country globally in the months of July and August 2020, due to its high per capita death rates in this period (4).

In this context, control mechanisms were created for each country (5). However, as in a company, the state learns based on what it knows and previous experience. Hence, many of these pandemic control mechanisms emerged along the way, and may not always be adequate (6). But also, as in a company, this should be seen retrospectively, so that they can serve as management indicators, which promote a “government audit” and, henceforth, generate plans for future similar situations (7).

Although the entire pandemic has generated multiple scenarios for action, the perception of the control mechanisms adopted by each government should be evaluated. This is especially true in a large context, such as Latin America, which has multiple realities such as varied epidemic evolution, populations with unequal reactions (8) and government decisions with different courses of action (9). Some data in this region has been reported. For example, a study that included data from Brazil and Colombia showed that people perceived an inadequate response of their governments compared to developed countries (10).

The perception of sanitary control during the pandemic may vary according to the particular characteristics of each population within a country. Understanding these characteristics could provide objective information on how the pandemic have affected the mental health of vulnerable groups, which may ultimately target prevention programs against mental disorders. For all these reasons, the objective of this study was to determine the factors associated with the perception of inadequate sanitary control in 12 Latin American countries during the COVID-19 pandemic.

## Materials and methods

### Design and population

This study is analytical cross-sectional because it was based on a single survey per person during June, July and August 2020. The data used here were collected in a previous study that aimed to assess the risk of PTSD caused by bereavement due to COVID-19 in 12 Latin American countries (Bolivia, Chile, Colombia, Costa Rica, Ecuador, El Salvador, Guatemala, Honduras, Mexico, Panama, Paraguay, and Peru). This primary study served the main author for a course within the doctorate in Economic Sciences (administration sub-area) at the University of Buenos Aires.

We included participants who predominantly resided in urban areas in any of the 12 Latin American countries during the months of execution. They expressed their willingness to participate in the research and responded to the main questions (the six about the perception of the government's sanitary control during the pandemic). We excluded eight minors, 1,973 respondents, as they did not answer all of the main questions, and 105 respondents residing in countries with low participation rates (Venezuela, Brazil, Argentina, and others).

It was calculated that there was an adequate statistical power based on the sample size. The amount of data was sufficient to be able to find differences in percentages of up to 2.5% (49 vs. 51.5%), since, with this, a statistical power of 90% was obtained. The sample was obtained non-randomly, with the support of a Peruvian network (Red COVID-19-GIS-Peru) and another in Latin America (FELSOCEM-ASOMEDISS COVID-19 Latam). We had the support of similar proportions of surveyors in both networks during the mentioned period, which meant that half of the responses were from Peru [the most affected country in the world (11)] and the other half from the rest of the countries.

### Variables and procedures

For the preparation of the questions, we considered the main perceptions that were expressed by political analysts and others who debated the way in which governments were dealing with the pandemic (12). It should be noted that these questions have been taken as exploratory, since they have not been subjected to a validation process “*per se*.” Thus, they basically responded to the most common questions that were obtained during the most critical moments of the first wave of COVID-19 in Latin America. These questions were distributed through the free Google Forms platform and, by using it, it was possible to reach the target population. They were distributed through the aforementioned networks.

The six items regarding the perception of government sanitary control were followed by the general question “How do you perceive these other things that have happened in these months? Responses were that (1) there was collective hysteria (i.e., emotional disturbance and fears related to the pandemic) in some sectors/moments, (2) there were shortages of basic goods, (3) there was a shortage of medicines, (4) the government could not/did not try to control the people, (5) the government could not/wanted to control the health sector, and (6) the government could not/did not manage to control the situation in general. These items were rated by respondents using a 5-point Likert scale, and ranged from 0 = Strongly disagree, to 4 = Strongly agree. Those who agreed or strongly agreed to the items were assumed to overall agree with the government's sanitary control.

Other variables were also used (in addition to the six questions and according to the area of residence in the 12 countries), such as educational level (primary, secondary, bachelor's degree, technical education, licentiate, or postgraduate studies), gender (male or female), age (years), not having a job at the time of the survey (yes or no) and having been diagnosed with COVID-19 (yes or no, according to self-report).

After collecting the questions, the questions were exported from the free platform to the Microsoft Excel program (for Windows), and then cleaned and analyzed.

### Data analysis

The statistical program Stata 11.1 (with a license) was used. The six questions of interest were evaluated in this program, where a Cronbach's Alpha of 0.90 was found (the individual Alpha values fluctuated between 0.88 and 0.91). All of them had a positive sign, adequate item-test correlations (between 0.75 and 0.88) and item-retest correlations (between 0.65 and 0.81). This process showed that the main questions were adequately understood by the surveyed population.

The descriptive analysis showed frequencies and percentages for categorical variables, and measures of central tendency and dispersion in the case of numerical variables. In addition, a stacked bar chart was generated for the six main questions, according to each of their five possible response options (strongly disagree, disagree, indifferent, agree and strongly agree). Then, each of the answers to the six main questions was dichotomized to see those who agreed with each of them (adding the strongly agree and agree categories) vs. the others (adding the indifferent, disagree and strongly disagree categories). Thus, we were able to obtain the percentage of those who agreed with each premise according to country of residence (we also obtained *p*-values with the chi-square test, to see if there was a difference among the answers of each country).

Then, multivariate analysis was performed to determine the factors associated with the perception of agreement with each of the six main premises. Generalized linear models, Poisson family, log link function and adjustment for robust variances were used. Prevalence ratios (PR) and 95% confidence intervals (95%CI) were estimated. For each of the cases, a *P*-value of < 0.05 was taken as statistically significant.

### Ethical approval

Ethical approval of the primary research was obtained from the ethics committee of Universidad Privada Antenor Orrego, Lima, Peru. It was not possible to obtain ethical approval from all Latin American countries because ethics committees were inoperative at the time of the study (due to the initial uncertainty generated by the COVID-19 pandemic). However, the Ethics Committee in Peru approved its development as a multicenter study. In addition, written consent was obtained after the study objective was explained to participants. Data were collected anonymously and remained confidential in all the research process.

## Results

Out of the 8,489 respondents throughout Latin America, the majority resided in Peru (53.8%), 41.1% were female and the median age was 22 years (18–89 years). Most of the respondents reported having a licentiate level (61.7%). Also, 70.6% reported that they were not working and had not been diagnosed with COVID-19 (97.7%).

**Figure 1:**
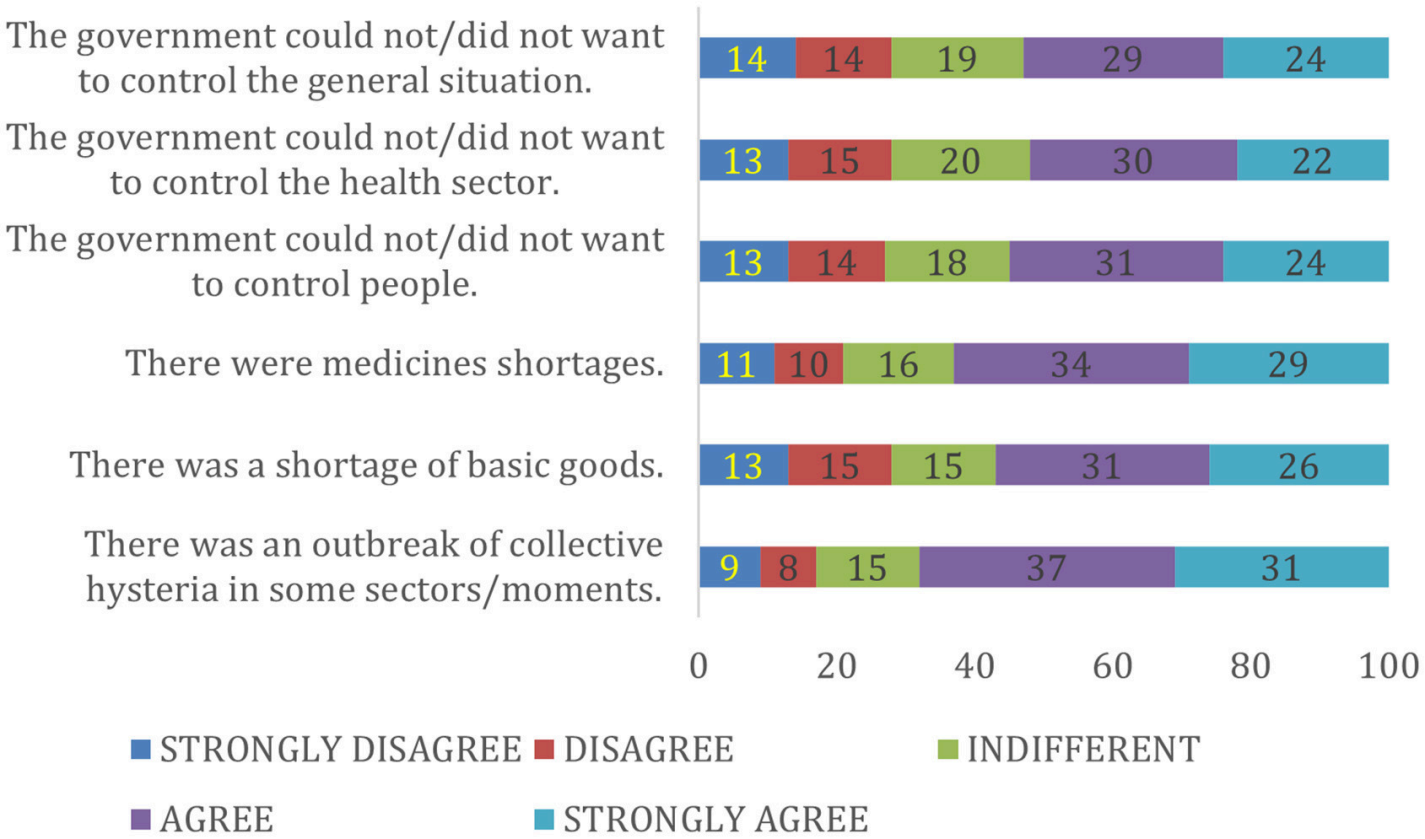
Perception of the six points of control mechanism in 12 Latin American countries during the first wave of the COVID-19 pandemic.

According to some degree of agreement, 68% stated that there were moments of collective hysteria (31% strongly agreed and 37% agreed), 63% claimed that there was a shortage of medicines (29% strongly agreed and 34% agreed) and 57% thought there was a shortage of basic essentials (26% strongly agreed and 31% agreed; Figure 1).

Regarding the perception that there was collective hysteria in some sectors/moments, the countries that perceived it the most were Honduras (80.2%) and Panama (81.0%), while those that perceived it the least were El Salvador (49.8%) and Guatemala (56.9%). In addition, regarding the perception of shortages of basic goods, those who perceived them the most were Panama (71.1%) and Honduras (74.6%); but those who perceived them the least were Chile (47.3%) and Costa Rica (44.8%). As for the perception of medicines shortages, those who perceived them the most were Ecuador (71.6%) and Honduras (79.6%); but those who perceived them the least were El Salvador (42.9%) and Costa Rica (30.7%; Table 1).

**Table 1:** Perception of collective hysteria and shortages in 12 Latin American countries during the first wave of the COVID-19 pandemic.

**Country**	**Collective hysteria in some sectors/ moments**	**Basic goods shortages**	**Medicines shortages**
Peru	65.3%	56.8%	67.8%
Chile	70.2%	47.3%	49.9%
Paraguay	72.3%	48.8%	54.7%
Mexico	69.0%	70.6%	66.4%
Colombia	76.2%	55.6%	57.1%
Bolivia	72.0%	52.8%	61.5%
Panama	81.0%	71.1%	64.3%
Ecuador	71.6%	62.2%	71.6%
Costa Rica	66.0%	44.8%	30.7%
El Salvador	49.8%	48.8%	42.9%
Honduras	80.2%	74.6%	79.7%
Guatemala	56.9%	49.0%	45.1%
Total	67.8%	56.5%	62.9%
*P*-value	<0.001	<0.001	<0.001

*P-values were obtained with the chi-square test*.

According to the perception of the control mechanisms that the government had regarding individuals, Costa Rica (27.4%) and Paraguay (35.2%) were those who perceived more control. On the other hand, Panama (66.9%) and Honduras (80.0%) were those who perceived less control. Regarding health system control, Costa Rica (13.7%) and El Salvador (32.5%) were those who perceived more control, while Ecuador (67.6%) and Honduras (79.7%) perceived less control. According to general control, Costa Rica (17.0%) and Paraguay (32.7%) were those who perceived more control, while Ecuador (69.0%) and Honduras (80.8%) perceived less control (Table 2).

**Table 2:** Inadequate control mechanisms that 12 Latin American governments had in three aspects during the first wave of the COVID-19 pandemic.

**Country**	**The government could not control people**	**The government could not control the health sector**	**The government could not control the general situation**
Honduras	80.0%	79.7%	80.8%
Panama	66.9%	58.3%	64.1%
Ecuador	66.6%	67.6%	69.6%
Colombia	64.3%	58.7%	57.9%
Chile	64.0%	56.3%	64.2%
Mexico	59.2%	53.9%	59.2%
Bolivia	57.7%	52.6%	54.9%
Peru	56.3%	53.2%	52.8%
El Salvador	37.9%	32.5%	37.4%
Guatemala	36.3%	38.2%	42.2%
Paraguay	35.2%	36.4%	32.7%
Costa Rica	27.4%	13.7%	17.0%
Total	55.7%	51.9%	53.3%
*P*-value	<0.001	<0.001	<0.001

*P-values were obtained with the chi-square test*.

When multivariate analysis was performed, it was found that there were statistically significant differences among many of the countries according to the perception of collective hysteria (eight statistical differences between countries: Chile, PR = 1.08, 95% CI = 1.03–1.14, Paraguay, PR = 1.08, 95% CI = 1.03–1.14, Colombia, PR = 1.13, 95% CI = 1.02–1.25, Bolivia, PR = 1.08, 95% CI = 1.02–1.15, Panama, PR = 1.22, 95% CI = 1.16–1.29, Ecuador, PR = 1.09, 95% CI = 1.01–1.18, El Salvador, PR = 0.76, 95% CI = 0.66–0.88, Honduras, PR = 1.18, 95% CI = 1.10–1.28), shortages of basic goods (seven statistical differences among countries: Chile, PR = 0.84, 95% CI = 0.78–0.91, Paraguay, PR = 0.83, 95% CI = 0.76–0.91, Mexico, PR = 1.20, 95% CI = 1.13–1.28, Panama, PR = 1.23, 95% CI = 1.15–1.32, Costa Rica, PR = 0.80, 95% CI = 0.70–0.93, El Salvador, PR = 0.86, 95% CI = 0.74–0.99, Honduras, PR = 1.25, 95% CI = 1.14–1.37) and medicines (eight statistical differences among countries: Chile, PR = 0.74, 95% CI = 0.69–0.79, Paraguay, PR = 0.80, 95% CI = 0.72–0.84, Colombia, PR = 0.84, 95% CI = 0.72–0.98, Bolivia, PR = 0.90, 95% CI = 0.83–0.97, Costa Rica, PR = 0.47, 95% CI = 0.38–0.57, El Salvador, PR = 0.63, 95% CI = 0.53–0.74, Honduras, PR = 1.13, 95% CI = 1.05–1.23, Guatemala, PR = 0.68, 95% CI = 0.55–0.84). In addition, the higher the academic degree, the greater the perceptions of inadequate control in the three aspects (for postgraduate studies: collective hysteria, PR = 1.48, 95% CI = 1.11–1.97, shortage of basic goods, PR = 1.83, 95% CI = 1.20–2.81, medicine shortages, PR = 1.46, 95% CI = 1.07–1.99). Men had a lower perception of inadequate control in all three aspects (collective hysteria: PR = 0.88, 95% CI = 0.86–0.92, shortage of basic goods: PR = 0.85, 95% CI = 0.82–0.89, medicine shortages: PR = 0.84, 95% CI = 0.81–0.87). The older the age, the lower the perception of inadequate control in terms of whether there was mass hysteria (PR = 1.00, 95% CI = 0.99–1.00) and shortages of basic essentials (PR = 0.99, 95% CI = 0.99–1.00). Those who had been diagnosed with COVID-19 had a lower perception of medicines shortages (PR = 0.86, 95% CI = 0.76–0.98; Table 3).

**Table 3:** Perception of collective hysteria and shortages in 12 Latin American countries during the first wave of the COVID-19 pandemic, in multivariate analysis.

**Country**	**Collective hysteria in some sectors/moments**	**Shortage of basic goods**	**Medicines shortages**
	**PR (95%CI)**	**PR (95%CI)**	**PR (95%CI)**
Country			
Peru	Comparison	Comparison	Comparison
Chile	1.08 (1.03–1.14)*	0.84 (0.78–0.91)*	0.74 (0.69–0.79)*
Paraguay	1.08 (1.03–1.14)*	0.83 (0.76–0.91)*	0.80 (0.72–0.84)*
Mexico	1.03 (0.97–1.10)	1.20 (1.13–1.28)*	0.96 (0.89–1.02)
Colombia	1.13 (1.02–1.25)*	0.96 (0.82–1.12)	0.84 (0.72–0.98)*
Bolivia	1.08 (1.02–1.15)*	0.91 (0.83–1.00)	0.90 (0.83–0.97)*
Panama	1.22 (1.16–1.29)*	1.23 (1.15–1.32)*	0.94 (0.87–1.02)
Ecuador	1.09 (1.01–1.18)*	1.07 (0.97–1.18)	1.06 (0.98–1.15)
Costa Rica	1.02 (0.92–1.13)	0.80 (0.70–0.93)*	0.47 (0.38–0.57)*
El Salvador	0.76 (0.66–0.88)*	0.86 (0.74–0.99)*	0.63 (0.53–0.74)*
Honduras	1.18 (1.10–1.28)*	1.25 (1.14–1.37)*	1.13 (1.05–1.23)*
Guatemala	0.90 (0.76–1.06)	0.91 (0.74–1.10)	0.68 (0.55–0.84)*
Education level			
Primary	Comparison	Comparison	Comparison
Secondary	1.55 (1.17–2.05)*	2.16 (1.42–3.28)*	1.58 (1.16–2.15)*
Bachelor's degree	1.52 (1.15–2.02)*	2.08 (1.36–3.17)*	1.54 (1.13–2.10)*
Technical education	1.47 (1.10–1.95)*	1.98 (1.30–3.03)*	1.48 (1.09–2.02)*
Licentiate	1.54 (1.17–2.04)*	2.03 (1.34–3.08)*	1.54 (1.13–2.08)*
Postgraduate studies	1.48 (1.11–1.97)*	1.83 (1.20–2.81)*	1.46 (1.07–1.99)*
Male gender	0.88 (0.86–0.92)*	0.85 (0.82–0.89)*	0.84 (0.81–0.87)*
Age (years)	1.00 (0.99–1.00)*	0.99 (0.99– 1.00)*	1.00 (1.00–1.00)
Unemployed	0.99 (0.95–1.03)	1.02 (0.97–1.08)	0.99 (0.94–1.03)
Diagnosed with COVID−19	0.92 (0.81–1.03)	0.88 (0.76–1.02)	0.86 (0.76–0.98)*

*P-values obtained through generalized linear models, Poisson family, log link function, and adjustment for robust variances*.

*P-values lower than 0.05.

When multivariate analysis was performed, it was found that there were statistically significant differences among many of the countries. Thus, these were according to the perception that the government did not know how to control people (eight statistical differences among countries: Chile, PR = 1.14, 95% CI = 1.08–1.21, Paraguay, PR = 0.61, 95% CI = 0.55–0.68, Panama, PR = 1.17, 95% CI = 1.08–1.26, Ecuador, PR = 1.17, 95% CI = 1.08–1.28, Costa Rica, PR = 0.50, 95% CI = 0.40–0.63, El Salvador, PR = 0.65, 95% CI = 0.54–0.80, Honduras, PR = 1.36, 95% CI = 1.25–1.47, Guatemala, PR = 0.65, 95% CI = 0.51–0.85), the health sector (six statistical differences among countries: Paraguay, PR = 0.67, 95% CI = 0.60–0.75, Ecuador, PR = 1.26, 95% CI = 1.16–1.38, Costa Rica, PR = 0.27, 95% CI = 0.19–0.38, El Salvador, PR = 0.60, 95% CI = 0.48–0.73, Honduras, PR = 1.43, 95% CI = 1.32–1.56, Guatemala, PR = 0.73, 95% CI = 0.57–0.93) or in general (eight statistical differences among countries: Chile, PR = 1.23, 95% CI = 1.16–1.30, Paraguay, PR = 0.61, 95% CI = 0.54–0.69, Mexico, PR = 1.11, 95% CI = 1.03–1.20, Panama, PR = 1.20, 95% CI = 1.11–1.31, Ecuador, PR = 1.32, 95% CI = 1.22–1.44, Costa Rica, PR = 0.33, 95% CI = 0.25–0.45, El Salvador, PR = 0.70, 95% CI = 0.58–0.84, Honduras, PR = 1.48, 95% CI = 1.36–1.60). In addition, when the academic level was higher, it was reported that there were higher perceptions of the government's inadequate control of people (for postgraduate: PR = 1.81, 95% CI = 1.20–2.73) or the health sector (for postgraduate: PR = 1.49, 95% CI = 1.00–2.20). Men had lower perceptions of inadequate control in all 3 aspects (inadequate control of people: PR = 0.90, 95% CI = 0.87–0.94, inadequate control of the health sector: PR = 0.91, 95% CI = 0.87–0.95, inadequate control of the general situation: PR = 0.93, 95% CI = 0.90–0.97; Table 4).

**Table 4:** Multivariate analytical statistics of the mechanism of inadequate control that 12 Latin American governments had in three aspects during the first wave of the COVID-19 pandemic.

**Country**	**The government could not control people**	**The government could not control the health sector**	**The government could not control the general situation**
Country			
Peru	Comparison	Comparison	Comparison
Chile	1.14 (1.08–1.21)*	1.06 (0.99–1.13)	1.23 (1.16–1.30)*
Paraguay	0.61 (0.55–0.68)*	0.67 (0.60–0.75)*	0.61 (0.54–0.69)*
Mexico	1.03 (0.95–1.12)	0.99 (0.91–1.08)	1.11 (1.03–1.20)*
Colombia	1.10 (0.96–1.26)	1.06 (0.91–1.24)	1.07 (0.91–1.25)
Bolivia	1.01 (0.92–1.10)	0.97 (0.88–1.07)	1.03 (0.94–1.13)
Panama	1.17 (1.08–1.26)*	1.08 (0.99–1.18)	1.20 (1.11–1.31)*
Ecuador	1.17 (1.08–1.28)*	1.26 (1.16–1.38)*	1.32 (1.22–1.44)*
Costa Rica	0.50 (0.40–0.63)*	0.27 (0.19–0.38)*	0.33 (0.25–0.45)*
El Salvador	0.65 (0.54–0.80)*	0.60 (0.48–0.73)*	0.70 (0.58–0.84)*
Honduras	1.36 (1.25–1.47)*	1.43 (1.32–1.56)*	1.48 (1.36–1.60)*
Guatemala	0.65 (0.51–0.85)*	0.73 (0.57–0.93)*	0.82 (0.65–1.03)
Education level			
Primary	Comparison	Comparison	Comparison
Secondary	1.91 (1.28–2.87)*	1.53 (1.04–2.25)*	1.35 (0.96–1.91)
Bachelor's degree	1.90 (1.26–2.85)*	1.53 (1.04–2.26)*	1.32 (0.93–1.86)
Technical	1.86 (1.24–2.80)*	1.51 (1.02–2.23)*	1.32 (0.93–1.87)
Licentiate	1.96 (1.31–2.93)*	1.60 (1.09–2.35)*	1.40 (1.00–1.97)
Postgraduate	1.81 (1.20–2.73)*	1.49 (1.00–2.20)*	1.29 (0.91–1.83)
Male gender	0.90 (0.87–0.94)*	0.91 (0.87–0.95)*	0.93 (0.90–0.97)*
Age (years)	1.00 (1.00–1.00)	1.00 (1.00–1.00)	1.00 (1.00–1.00)
Unemployed	1.04 (0.98–1.09)	1.02 (0.96–1.08)	1.01 (0.96–1.07)
COVID-19 positive	0.91 (0.79–1.04)	0.99 (0.87–1.14)	1.01 (0.89–1.15)

*P-values were obtained through generalized linear models, Poisson family, log link function, and adjustment for robust variances*.

*P-values lower than 0.05.

## Discussion

Most of the respondents answered that they agreed that there were moments of mass hysteria. This was corroborated by multiple journalistic reports (13–15), where it was shown that some near and far countries have perceived these scenes. Collective hysteria was reported especially in the first moments of the pandemic, where chaos took place in some cities. That was intensified by the news that were available through various media, which even generated a sort of “infodemic” or pandemic of misinformation (16, 17). Therefore, it is not strange that this was perceived by the recruited sample, since chaos, anarchy and general lack of control were the most feared repercussions by society (18). Unfortunately, there were no scientific reports of this; hence, it is important to study it in depth, as this could generate later sequelae, such as post-traumatic stress.

The sample also perceived, to a great extent, the fact that there was a shortage of medicines and basic essentials at specific times of the pandemic. These shortages of basic goods were perceived in the early stages, when many governments decided to create restrictions and quarantines (19–21). But this also occurred when the quarantine began to be prolonged, since many of those in the poorer classes did not have sufficient resources to be able to take food home (22). On the other hand, the shortage of medicines occurred after the announcement that a drug was likely to help in the treatment. This was the case of hydroxychloroquine (23, 24), azithromycin (25), ivermectin (26), and among many others. Thus, the announcement of their possible effectiveness caused people to go out and buy these medicines, which generated a momentary shortage (27). This problem with medicines could also be perceived by families who had one or more relatives with COVID-19, which caused them to urgently require various medicines that were necessary at the time (oxygen, for example) (28).

As for the countries that most perceived inadequate control mechanisms, the one that stands out the most was Honduras since this country has extremely high percentages of the six perceptions. This could be due to the multiple health and economic problems that this country was already facing before the pandemic (29, 30). This situation was exacerbated when the disease arrived in this territory, which generated a lot of chaos and repercussions in every family and individual, especially due to the precariousness of its health system, which collapsed when the virus arrived (17, 31). Of course, this also happened in many other Latin American countries as several reports show that these problems occurred in different countries of our continent (32). It is also important to mention that some countries had a lower perception of inadequate control, such as Costa Rica and Panama. It was probable because these countries still maintain very close relations with first world countries, which for several decades have influenced a better form of internal administration (33).

According to what was mentioned in the previous paragraph, in addition to what was obtained in the multivariate analysis, it was found that there were very marked differences among most countries according to each of the perceptions of inadequate control. They were adjusted by important characteristics of the population. Thus, these differences were expected since each population has had different perceptions of the management of the pandemic over time (33, 34). Latin American governments had similarities in the management of the pandemic, such as the mandatory use of face masks, mass screening of COVID-19 cases, and quarantine. However, some countries had a timely response compared to others (35). For example, Argentina, Colombia, Ecuador, Guatemala, and Paraguay has built hospitals in few days to provide additional ICU beds. Costa Rica approved guidelines for the management of mental health problems associated with the pandemic. Costa Rica, El Salvador, and Peru implemented phone call services to manage emergencies and deliver medicines. Chile and Uruguay established innovative and massive screening strategies to better identify COVID-19 cases. However, as material and human resources were depleted, the false perception of control of the pandemic faded (36). Many countries in our region had truly terrifying figures; for example, Peru became for many weeks the country with the highest mortality per million inhabitants worldwide (37).

Compared to those with primary education (or a lower level), those with higher levels of schooling perceived a worse management of the situation. This has been described in other research, where those who are professionals from wealthy social classes and other circles are the ones who demand order and good government management the most, among many other demands (38, 39). This does not mean that lower social classes have a lower standard, but many times these groups are used to subsisting on informality, precarious conditions, low social and health coverage, among many other precariousness that have been reported since before the pandemic (40). It could also be influenced by the fact that the higher the socio-economic level, the more quality is demanded in all aspects of life. This has not been studied in our research, as well as many other important social variables that could be influencing the found differences (41). However, these should be evaluated in other research that combines epidemiological and social aspects.

Men had a lower perception of inadequate control regarding the six evaluated criteria. This has been researched in multiple studies from different perspectives. One of the most important is the fact that women are the ones who are most concerned about the stability of the household due to their role as the ones in charge of bringing food and covering basic needs. Therefore, if there is something that can alter this, women will be the ones whose mental state will be most affected (42, 43). It could also be because multiple studies place women as the ones who have more anxiety, stress, depression, and other mental conditions (44). Hence, they will be much more affected by having these global problems. This generates multiple interventions, which take female sex as one of the most vulnerable groups, and even more if we add that they have other characteristics of social abandonment (44).

The older the age, the lower the perception of inadequate control in terms of collective hysteria and shortage of basic goods. This could be influenced by the greater saturation generated by social media, especially those that are more accessible to young people, which not only transmitted a lot of false information, but also caused an increase in fear in various communities (45). Another aspect that could have influenced youngsters was the fact that they have a lower tolerance toward confinement, quarantine or following the rules. Moreover, due to their age, they like parties, social gatherings and other activities that were banned since the beginning of the pandemic. Consequently, this increases their levels of stress, anxiety, and others (46–48). It could also be due to a lower understanding of the news given by the media, which has been widely reported in recent years, especially in developing countries, where it has been shown that the levels of understanding are very poor (49, 50).

Finally, those who were diagnosed with COVID-19 had a lower perception that there was a shortage of medicines. This result is interesting because, although the population generally perceived that there was a medicines shortage, those who became infected with the disease had the opposite perception. This could be explained because before getting infected many people had fatalistic thoughts (that they were going to die, that they were going to be admitted to the intensive care unit, among others) (51). However, once they got sick many of them confirmed that their illness was not as serious as it appeared in the media and that symptomatic treatment is often sufficient (52, 53). Another possible explanation could be the fact that, although there were many problems to obtain medicines at certain times, some of the governments put strict rules, gave support, and tried their best to implement their health system to cope with the pandemic (54). Other reasons respondents had should be addressed in future research.

### Limitations

It is important to mention that the research had some methodological limitations; an important one is the fact that the results cannot be extrapolated to the entire population of Latin America, because the type of the used sampling was non-random. Another limitation is that the survey was predominantly conducted with people residing in urban areas, especially in the main capitals of each country. However, a large population was surveyed in this region of the Americas, much larger than other investigations, which may be a clear indicator of what urban populations and those residing in large cities think. In addition, multivariate analysis allows us to generate hypotheses about the possible causes of the population's negative perception of government sanitary control. It is worth mentioning that the large number of respondents improves the validity of the inference analyses. This provides useful information for governments on the effectiveness of epidemic control from the point of view of their citizens. Overall, the results shown here should be considered preliminary; hence, future research should try to investigate more on the subject, with a larger population, with a greater number of variables and even trying to make a research that is more in line with the social aspect.

## Conclusion

Based on the above, it can be concluded that what was perceived most was collective hysteria. Honduras was the country that most perceived inadequate sanitary control mechanisms established by the government. There were important differences among many of the countries according to the six evaluated items. The higher the level of education, the greater the perception of poor control in five of the items. Men had a lower perception of inadequate sanitary control in five aspects. The older the age, the lower the perception of control in two of the aspects. Those who were diagnosed with COVID-19 had a lower perception that there were medicines shortages.

The implication of this study is that urban populations and people residing in large cities generally perceive that their governments negatively perform sanitary control. Therefore, it is undeniable that their perception is an indicator of the performance of each government during the COVID-19 pandemic. Studying the problem of sanitary control in this context could provide new information on the relationship between the effectiveness of government sanitary control and people's mental health, which ultimately helps to create objective prevention programs against post-traumatic stress disorder, depression, fear of contagion, and collective hysteria. In addition, for governments these findings could serve as objective indicators to build mitigation plans for future unavoidable pandemic events based on the six criteria discussed here.

## Data availability statement

The raw data supporting the conclusions of this article will be made available by the authors, without undue reservation.

## Ethics statement

The studies involving human participants were reviewed and approved by Ethics Committee of Universidad Privada Antenor Orrego, Lima, Peru. The patients/participants provided their written informed consent to participate in this study.

## Author contributions

CM: conception and design of the work, acquisition, analysis, and interpretation of data, drafted the work and revised it critically, and approved the version to be published. DL-V: analysis and interpretation of data, revised the work critically, and approved the version to be published. FG-G and MM-R: interpretation of data, drafted the work and revised it critically, and approved the version to be published. MV-G: design of the work, analysis and interpretation of data, revised the work critically, and approved the version to be published. All authors contributed to the article and approved the submitted version.

## Funding

MV-G has received support by the Fogarty International Center of the National Institutes of Mental Health (NIMH) under Award Number D43TW009343 and the University of California Global Health Institute. The funders had no role in study design, data analysis, decision to publish, or preparation of the manuscript.

## Conflict of interest

The authors declare that the research was conducted in the absence of any commercial or financial relationships that could be construed as a potential conflict of interest.

## Publisher's note

All claims expressed in this article are solely those of the authors and do not necessarily represent those of their affiliated organizations, or those of the publisher, the editors and the reviewers. Any product that may be evaluated in this article, or claim that may be made by its manufacturer, is not guaranteed or endorsed by the publisher.

## References

[1] UNESCO. COVID-19: Problemas sociales y psicológicos en la pandemia. (2020). Available online at: https://es.unesco.org/news/covid-19-problemas-sociales-y-psicologicos-pandemia (accessed February 4, 2021).

[2] BBC News Mundo. Cómo cambió el mundo hace cien años con la gripe española, la peor pandemia del siglo XX. (2020). Available online at: https://www.bbc.com/mundo/noticias-52473180 (accessed February 4, 2021).

[3] Peñafiel-Chang L Camelli G Peñafiel-Chang P. Pandemia COVID-19: Situación política - económica y consecuencias sanitarias en América Latina: Cienc. UNEMI. (2020) 13:120–8. 10.29076/issn.2528-7737vol13iss33.2020pp120-128p

[4] El Comercio. Por qué Perú es el país con la mayor tasa de mortalidad entre los más afectados por la pandemia. (2020). Available online at: https://elcomercio.pe/tecnologia/ciencias/covid-19-por-que-peru-es-el-pais-con-la-mayor-tasa-de-mortalidad-entre-los-mas-afectados-por-la-pandemia-noticia/ (accessed February 4, 2021).

[5] UNDP. Un abordaje integral para responder a la COVID-19 en Perú. (2020). Available online at: https://www.undp.org/content/undp/es/home/blog/2020/peru-embarks-on-a-comprehensive-approach-to-covid-19.html (accessed February 4, 2021).

[6] Mejia CR Rodriguez-Alarcon JF Garay-Rios L Enriquez-Anco MG Moreno A Huaytán-Rojas K. Percepción de miedo o exageración que transmiten los medios de comunicación en la población peruana durante la pandemia de la COVID-19. Rev Cuba Investig Bioméd. (2020) 39:e698E. Available online at: http://scielo.sld.cu/scielo.php?script=sci_arttext&pid=S0864-03002020000200001

[7] Gobierno del Perú. Gobierno anuncia nuevas medidas para frenar contagios por COVID-19. (2020). Available online at: https://www.gob.pe/institucion/minsa/noticias/324674-gobierno-anuncia-nuevas-medidas-para-frenar-contagios-por-covid-19 (accessed February 4, 2021).

[8] Araujo-Banchon WJ Aveiro-Róbalo TR Fernández MF Castro-Pacoricona D Moncada-Mapelli E Chanava W. Progresión de casos de Coronavirus en Latinoamérica: Análisis comparativo a una semana de iniciada la pandemia en cada país. Kasmera. (2020) 48:e48131621. 10.5281/zenodo.3830750

[9] AS/COA. El coronavirus en América Latina. (2020). Available online at: https://www.as-coa.org/articles/el-coronavirus-en-america-latina (accessed February 4, 2021).

[10] Mækelæ MJ Reggev N Dutra N Tamayo RM Silva-Sobrinho RA Klevjer K. Perceived efficacy of COVID-19 restrictions, reactions and their impact on mental health during the early phase of the outbreak in six countries. R Soc Open Sci. (2020) 7:200644. 10.1098/rsos.200644 32968525 PMC7481706

[11] Díaz-Vélez C Failoc-Rojas VE Valladares-Garrido MJ Colchado J Carrera-Acosta L Becerra M. SARS-CoV-2 seroprevalence study in Lambayeque, Peru. June-July 2020. PeerJ. (2021) 9:e11210. 10.7717/peerj.11210 33868828 PMC8034367

[12] Quiroz Carrillo CG Pareja Cruz A Valencia Ayala E Enriquez Valencia YP De Leon Delgado J Aguilar Ramirez P. Un nuevo coronavirus, una nueva enfermedad: COVID-19. Horiz Méd Lima. (2020) 20:11. 10.24265/horizmed.2020.v20n2.11

[13] TVN. Recomiendan establecer horarios para actividades recreativas y cuidar la salud mental de jóvenes. (2021). Available online at: https://www.tvn-2.com/nacionales/Recomiendan-establecer-horarios-actividades-recreativas-cuidar-salud-mental-de-jovenes-panama_0_5762423718.html (accessed February 4, 2021).

[14] Tiempo. Web R. Médico Osmín Tovar: La histeria colectiva hace más daño que el Covid-19. (2020). Available online at: https://tiempo.hn/medico-osmin-tovar-la-histeria-colectiva-hace-mas-dano-que-el-covid-19/ (accessed February 4, 2021).

[15] Dirección General de Comunicación Social – UNAM. Además de pandemia por COVID-19, México enfrenta propagación de noticias falsas. (2020). Available online at: https://www.dgcs.unam.mx/boletin/bdboletin/2020_318.html (accessed February 4, 2021).

[16] COVID-19 Honduras. Comunicado suspensión de fase 1 en el Distrito Central por incumplimiento. (2020). Available online at: https://covid19honduras.org/?q=suspension-fase-1-distrito-central (accessed February 4, 2021).

[17] BBC News Mundo. Coronavirus en Honduras: la polémica compra de clínicas móviles para afrontar la pandemia (que han tardado meses en estar operativos). (2020) Available online at: https://www.bbc.com/mundo/noticias-america-latina-54548236 (accessed February 4, 2021).

[18] Consejo Nacional Anticorrupción Honduras. Informe de prácticas erróneas en la administración pública. (2020). Available online at: https://www.cna.hn/2020/09/14/informe-de-practicas-erroneas-en-la-admon-publica/ (accessed February 4, 2021).

[19] COVID-19 Honduras. Se declara toque de queda a nivel nacional y absoluto para el Distrito Central. (2020) Available online at: https://covid19honduras.org/?q=toque-de-queda (accessed February 4, 2021).

[20] El Comercio. Coronavirus en Perú: Martín Vizcarra decretó Estado de Emergencia Nacional y aislamiento social para evitar contagios de COVID-19. (2020) Available online at: https://elcomercio.pe/lima/coronavirus-en-peru-martin-vizcarra-anuncia-cuarentena-general-por-15-dias-para-evitar-mas-contagios-covid-19-pandemia-nndc-noticia/ (accessed February 4, 2021).

[21] BBC News Mundo. Cómo hace frente al COVID-19 cada país de América Latina. (2020). Available online at: https://www.bbc.com/mundo/noticias-america-latina-51881075 (accessed February 4, 2021).

[22] PNUD en América Latina y El Caribe. La seguridad alimentaria frente a la pandemia del COVID-19. (2020). Available online at: https://www.latinamerica.undp.org/content/rblac/es/home/blog/2020/la-seguridad-alimentaria-frente-a-la-pandemia-del-covid-19.html (accessed February 4, 2021).

[23] Gao J Tian Z Yang X. Breakthrough: Chloroquine phosphate has shown apparent efficacy in treatment of COVID-19 associated pneumonia in clinical studies. Biosci Trends. (2020) 14:72–3. 10.5582/bst.2020.01047 32074550

[24] Yao X Ye F Zhang M Cui C Huang B Niu P. *In vitro* antiviral activity and projection of optimized dosing design of hydroxychloroquine for the treatment of severe acute respiratory syndrome coronavirus 2 (SARS-CoV-2). Clin Infect Dis. (2020) 71:732–9. 10.1093/cid/ciaa237 32150618 PMC7108130

[25] Damle B Vourvahis M Wang E Leaney J Corrigan B. Clinical pharmacology perspectives on the antiviral activity of azithromycin and use in COVID-19. Clin Pharmacol Ther. (2020) 108:201–11. 10.1002/cpt.1857 32302411 PMC7262099

[26] Caly L Druce JD Catton MG Jans DA Wagstaff KM. The FDA-approved drug ivermectin inhibits the replication of SARS-CoV-2 *in vitro*. Antiviral Res. (2020) 178:104787. 10.1016/j.antiviral.2020.104787 32251768 PMC7129059

[27] Lupus Research. La Alianza para la investigación de Lupus pone en perspectiva la escasez de Plaquenil (hidroxicloroquina) para la comunidad con lupus. (2020). Available online at: https://www.lupusresearch.org/declaracion-del-26-de-marzo-la-alianza-para-la-investigacion-de-lupus-pone-en-perspectiva-la-escasez-de-plaquenil-hidroxicloroquina-para-la-comunidad-con-lupus/ (accessed February 4, 2021).

[28] BBC News Mundo. Cómo la COVID-19 está causando una ≪crisis de oxígeno≫ en América Latina y algunos países en desarrollo. (2021). Available online at: https://www.bbc.com/mundo/noticias-internacional-55841858 (accessed February 4, 2021).

[29] Welle D. Corrupción hunde a Honduras en la pobreza y la desigualdad. (2020). Available online at: https://www.dw.com/es/corrupci%C3%B3n-hunde-a-honduras-en-la-pobreza-y-la-desigualdad/a-52565026 (accessed February 4, 2021).

[30] Comisión Económica para América Latina y El Caribe. Sistemas de protección social en América Latina y El Caribe: Honduras. (2013). Available online at: https://repositorio.cepal.org/handle/11362/4069 (accessed February 4, 2021).

[31] Diario La Prensa. El tórax alcanza su máxima capacidad de pacientes COVID-19. (2020). Available online at: https://www.laprensa.hn/honduras/1431884-410/hospital-el-torax-capacidad-pacientes-covid- (accessed February 4, 2021).

[32] The New York Times. Ecuador: la cantidad de muertos por coronavirus está entre las peores del mundo. (2020). Available online at: https://www.nytimes.com/es/2020/04/23/espanol/america-latina/virus-ecuador-muertes.html (accessed February 4, 2021).

[33] BBC News Mundo. Cuál es la efectiva fórmula contra el coronavirus de Costa Rica, el país de América Latina donde mueren menos pacientes de COVID-19. (2020). Available online at: https://www.bbc.com/mundo/noticias-america-latina-52480615 (accessed February 4, 2021).

[34] The New York Times. El coronavirus solo es uno de muchos brotes en estos países. (2020). Available online at: https://www.nytimes.com/es/2020/06/01/espanol/america-latina/dengue-honduras-virus.html (accessed February 4, 2021).

[35] OECD. Covid-19 en América Latina y el Caribe: Panorama de las respuestas de los gobiernos a la crisis. Paris: OECD (2020). Available online at: https://www.oecd-ilibrary.org/economics/covid-19-en-america-latina-y-el-caribe-panorama-de-las-respuestas-de-los-gobiernos-a-la-crisis_7d9f7a2b-es (accessed June 12, 2022).

[36] BBC News Mundo. Cómo el coronavirus está poniendo a prueba el poder ≪faraónico≫ de Putin en Rusia. (2020). Available online at: https://www.bbc.com/mundo/noticias-internacional-52377955 (accessed February 4, 2021).

[37] BBC News Mundo. Razones por las que Perú tiene la mayor tasa de mortalidad entre los países más afectados por el coronavirus. (2020). Available online at: https://www.bbc.com/mundo/noticias-america-latina-53940042 (accessed February 4, 2021).

[38] Instituto Nacional de Estadística e Informática. Perú: Percepción Ciudadana sobre Gobernabilidad, Democracia y Confianza en las Instituciones: Octubre 2019 - Marzo 2020. (2020). Available online att: http://m.inei.gob.pe/biblioteca-virtual/boletines/gobernabilidad-democracia-y-confianza-en-las-instituciones-9866/1/#lista (accessed February 4, 2021).

[39] Miranda GB. Fracturados, pero no rotos. (2020). Radio Programas del Perú. Available online at: https://rpp.pe/columnistas/gisellabenavente/fracturados-pero-no-rotos-noticia-1299474 (accessed February 4, 2021).

[40] Sartorius N. Poverty and health. Croat Med J. (2007) 48:750–1. 10.3325/cmj.2007.6.878 17948962 PMC2205979

[41] World Health Organization. El nivel socioeconómico prevalece sobre la edad y el sexo como determinante de la búsqueda de atención sanitaria en el Bangladesh rural. (2005). Available online at: https://www.who.int/bulletin/volumes/83/2/ahmed0205abstract/es/ (accessed February 4, 2021).

[42] World Health Organization. Gender and mental health. (2002). Available online at: https://www.who.int/gender-equity-rights/knowledge/a85573/en/ (accessed February 4, 2021).

[43] Afifi M. Gender differences in mental health. Singapore Med J. (2007) 48:385–91. 17453094

[44] Connor J Madhavan S Mokashi M Amanuel H Johnson NR Pace LE. Health risks and outcomes that disproportionately affect women during the Covid-19 pandemic: A review. Soc Sci Med. (2020) 266:113364. 10.1016/j.socscimed.2020.113364 32950924 PMC7487147

[45] Patel MP Kute VB Agarwal SK. “Infodemic” COVID 19: more pandemic than the virus. Indian J Nephrol. (2020) 30:188–91. 10.4103/ijn.IJN_216_20 33013069 PMC7470201

[46] World Health Organization. Orientaciones para el público. (2020). Available online at: https://www.who.int/es/emergencies/diseases/novel-coronavirus-2019/advice-for-public (accessed February 4, 2021).

[47] Nivette A Ribeaud D Murray A Steinhoff A Bechtiger L Hepp U. Non-compliance with COVID-19-related public health measures among young adults in Switzerland: insights from a longitudinal cohort study. Soc Sci Med. (2021) 268:113370. 10.1016/j.socscimed.2020.113370 32980677 PMC7493799

[48] Jia R Ayling K Chalder T. Young people, mental health and COVID-19 infection: the canaries we put in the coal mine. Public Health. (2020) 189:158–61. 10.1016/j.puhe.2020.10.018 33249392 PMC7598559

[49] Bao Y Sun Y Meng S Shi J Lu L. 2019-nCoV epidemic: address mental health care to empower society. Lancet. (2020) 395:e37–8. 10.1016/S0140-6736(20)30309-3 32043982 PMC7133594

[50] El Comercio. Prueba Pisa 2018: Perú ocupa puesto 64 de 77 países evaluados. (2019). Available online at: https://elcomercio.pe/peru/prueba-pisa-peru-ocupa-puesto-64-de-77-paises-segun-ultimo-reporte-nndc-noticia/ (accessed February 4, 2021).

[51] Centers for Disease Control Prevention. El COVID-19 y su salud. (2020). Available online at: https://espanol.cdc.gov/coronavirus/2019-ncov/need-extra-precautions/older-adults.html (accessed February 4, 2021).

[52] World Health Organization. Consejos para la población acerca de los rumores sobre el nuevo coronavirus (2019-nCoV). (2020). Available online at: https://www.who.int/es/emergencies/diseases/novel-coronavirus-2019/advice-for-public/myth-busters (accessed February 4, 2021).

[53] Umakanthan S Sahu P Ranade AV Bukelo MM Rao JS Abrahao-Machado LF. Origin, transmission, diagnosis and management of coronavirus disease 2019 (COVID-19). Postgrad Med J. (2020) 96:753–8. 10.1136/postgradmedj-2020-138234 32563999 PMC10016932

[54] Food Drug Administration. FDA Revokes Emergency Use Authorization for Chloroquine and Hydroxychloroquine. (2020). Available online at: https://www.fda.gov/news-events/press-announcements/coronavirus-covid-19-update-fda-revokes-emergency-use-authorization-chloroquine-and (accessed February 4, 2021).

